# Targeting the invincible barrier for drug delivery in solid cancers: interstitial fluid pressure

**DOI:** 10.18632/oncotarget.26267

**Published:** 2018-11-06

**Authors:** Steven K. Libutti, Lawrence Tamarkin, Naris Nilubol

**Affiliations:** ^1^ Department of Endocrine Oncology Branch, NCI/NIH, Bethesda, MD, USA

**Keywords:** microenvironment, interstitial fluid pressure, drug delivery, tumor necrosis factor, nanomedicine

## Abstract

Although a number of new systemic therapeutic options in patients with advanced solid cancers have emerged due to the improved knowledge of molecular dysregulation in cancers, the durable, long-term, objective responses infrequently occur. This editorial article highlights the major limitation of current systemic therapy due to an inefficient drug delivery. While several mechanisms contributing to cancer drug resistance have been described, the common key barrier among solid cancers is the unique tumor microenvironment that causes the high interstitial fluid pressure (IFP). We discussed the mechanism causing an elevated IFP and how it interferes with drug delivery. To target the high IFP, we demonstrated the novel approach using gold nanoparticle carrying recombinant human tumor necrosis factor (TNF), a vascular disrupting agent, that preferentially and specifically targets tumors while the systemic toxicity is markedly reduced. The addition of cytotoxic agent by either directly conjugating to the gold nanoparticle or by systemic administration following gold nanoparticle carrying TNF resulted in significantly reduced tumor burden and increased survival in multiple mouse models with primary and metastatic endocrine cancer and pancreatic ductal carcinoma. A clinical trial in patients with advanced solid cancers is warranted based on the promising results in preclinical studies.

In the era of “precision medicine” for cancer, efforts have been made to identify agents that preferentially target expressed molecular pathways specific to the cancer cells. Despite the potential of these new targeted therapies, durable long-term responses rarely occur. A major limitation of current systemically delivered cancer therapies is the failure of these drugs to effectively reach their target, the cancer cells. Because of inefficient systemic drug delivery, high doses of systemically-administered cancer drugs are usually given, which inevitably cause off-target toxicity, to achieve anti-tumor efficacy. Several factors contribute to the failure of systemic cancer treatments to effectively kill cancer cells. These include the ATP-binding cassette (ABC) transporters that can actively pump cytotoxic drugs out of cancer cells [1] and the program death ligand 1 on tumor cells which allows cancer cells to evade immune system detection [2]. However, the high interstitial fluid pressure (IFP) in the tumor microenvironment is the key barrier commonly seen in solid cancers that has not been widely considered and that can impede therapeutic drug delivery to tumors. Elevated tumor IFP in the tumor microenvironment is believed to be from high cell density, increased intratumoral vascular permeability, abnormal extracellular matrix, and poor venous/lymphatic drainage [3].

Normally, water and molecules transported through convection flow out of capillaries, flow through the interstitial space, and into the lymphatic system and/or the venous drainage system. This transcapillary flow, created by the gradients of hydrostatic and colloid osmotic pressures in capillaries and in the interstitium, is typically outward (capillaries to lymphatic and/or venous system) because of the slightly negative pressure gradients in the normal interstitium [4]. The elevated tumor IFP markedly reduces drug delivery efficacy due to a drop of convection between the intra- and extra-vascular spaces, thus limiting drug distribution into the tumor microenvironment. The increased IFP has been reported in many solid tumors, such as breast, colorectal cancers, and melanoma [4] and has been associated with a poor prognosis in melanoma and cervical cancer due to lower treatment response [4, 5]. In preclinical models, Ferretti et al. [6] demonstrated that tumor IFP can be reduced when tumor-burdened mice with melanoma and breast cancer were treated with a vascular disrupting agent. The reduction in IFP correlated well with tumor size reduction [6]. By targeting components of the tumor microenvironment responsible for creating high IFP, drug delivery to tumors can be improved, potentially leading to improved patient outcomes.

Tumor necrosis factor alpha (TNF) is a cytokine involved in systemic inflammatory response. This cytokine also has a potent effect on vascular endothelium, causing vascular leakage. Because of the severe systemic toxicity, the EU-approved clinical role for TNF in cancer treatment is currently limited to a cumbersome but effective surgical procedure that regionally delivers the combination of TNF and a high dose cytotoxic agent to the affected limb for locally advanced melanoma (isolated limb perfusion). To mitigate the systemic toxicity associated with TNF, our group demonstrated in a phase I clinical trial in patients with advanced solid cancers that CYT-6091, a PEGylated colloidal gold nanoparticle carrying recombinant human TNF, preferentially targets tumor tissue and had no dose-limiting toxicity at a dose 3-times higher than previous clinical trials in cancer patients treated with systemically administered recombinant human TNF. Although CYT-6091 was safe, the anti-tumor activity was minimal as only one patient of 27 achieved a partial response [7]. In a number of preclinical models, we demonstrated that CYT-6091 significantly reduced tumor IFP, caused vascular endothelial damage and leakage with synergistic antitumor activity when combined with radiation in mice with breast cancer [8]. Thus, CYT-6091 must be used in combination with other cytotoxic agents to achieve a meaningful anti-tumor response.

To address this limitation, a second generation PEGylated colloidal gold nanoparticle was created. CYT-21625 is a PEGylated colloidal gold nanoparticle carrying recombinant human TNF *and* a paclitaxel prodrug. We studied the effect of CYT-21625 and CYT-6091 in 3 mouse models of thyroid cancer and neuroendocrine tumors. In this recent publication, we performed CT scan, FDG-PET, and histologic analyses showing that CYT-6091 and CYT-21625 specifically and preferentially target tumor tissue without histologic or clinical evidence of toxicity to normal organs. In addition, both nanomedicines caused intratumoral vascular damage and leakage only in tumor tissue. CYT-21625 treatment resulted in statistically significant lower tumor burden in mice with metastatic anaplastic thyroid cancer, poorly differentiated thyroid cancer (PDTC) and in genetically engineered mice that naturally develop pancreatic neuroendocrine tumors. The survival benefit was observed in mice with metastatic PDTC. Histologic analysis showed that CYT-21625 treated xenografts had lower tumor cell proliferation and increased caspase-dependent apoptosis [9]. For the first time, the combination of a cytotoxic drug and a vascular disrupting agent targeting IFP on a nanoparticle delivery system was shown to be effective in solid cancers with no detectable toxicity. These findings confirm that by reducing tumor IFP, the anti-tumor efficacy of cytotoxic drug increases. Next, we hypothesized that a pre-treatment of the tumor microenvironment using CYT-6091 followed by systemically administered paclitaxel can result in significantly improved anti-tumor efficacy. Genetically engineered mice with pancreatic adenocarcinoma were treated weekly with CYT-6091 3-hours before the administration of protein-bound paclitaxel. Fifty percent of mice receiving protein-bound paclitaxel only survived at day 42 post-treatment while *all* mice that received CYT-6091 pretreatment followed by protein-bound paclitaxel survived. None of the mice receiving vehicle control survived at day 42 (Figure 1). Our results suggest that a pretreatment with CYT-6091 can improve the outcome of systemically administered cytotoxic agent(s). This treatment strategy can be applicable to patients with a wide range of solid cancers receiving currently-approved therapies.

**Figure 1:**
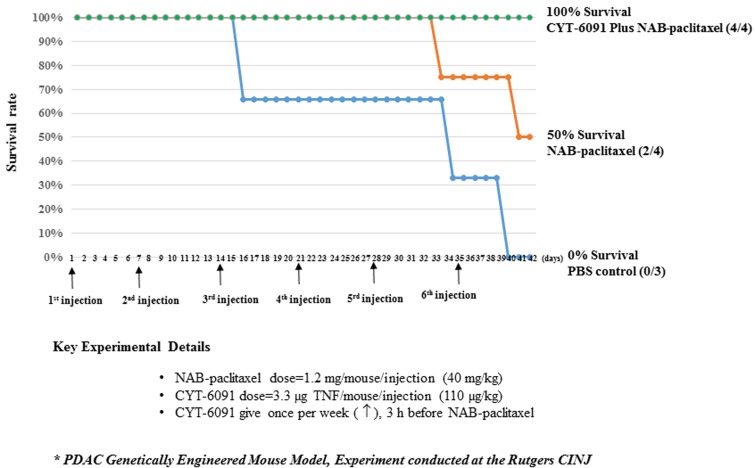
The survival rate of genetically engineered mice with pancreatic ductal adenocarcinoma treated with a weekly injection of nano-albumin bound paclitaxel (NAB-paclitaxel) alone (orange line, *n* = 4), NAB-paclitaxel 3 hours after CYT-6091 treatment (green line, *n* = 4), and vehicle control (blue line, *n* = 3) The experiment was performed at the Rutgers Cancer Institute of New Jersey. The doses of paclitaxel and TNF were 40 mg/kg and 110 µg/kg, respectively. Arrows indicate the day of treatments.

In summary, the unique characteristics of the microenvironment in solid cancers such as highly permeable tumor vasculature contribute to high IFP causing ineffective drug delivery and treatment failure. Targeting tumor vasculature with TNF to optimize the tumor microenvironment increases the efficacy of the cytotoxic agent, either carried on the nanomedicine platform or administered separately. This approach provides a unique opportunity to improve drug delivery to solid cancers and treatment outcome. Preclinical studies of systemically administered CYT-21625 and CYT-6091 followed by the cytotoxic agent in genetically engineered mouse models that spontaneously develop solid tumors (pancreatic neuroendocrine tumors or pancreatic ductal adenocarcinoma) support this approach as the treatments induced tumor vascular leakage, increased cytotoxic drug accumulation, and improved animal survival. Because tumor vascular architecture in these mice resembles that seen in patients, such promising responses in preclinical studies of nanomedicine targeting tumor microenvironment need to be expanded to patients with advanced solid cancers in clinical trials.
